# Aggregation induced emission based active conjugated imidazole luminogens for visualization of latent fingerprints and multiple anticounterfeiting applications

**DOI:** 10.1038/s41598-021-96011-5

**Published:** 2021-08-18

**Authors:** M. K. Ravindra, G. P. Darshan, D. R. Lavanya, K. M. Mahadevan, H. B. Premkumar, S. C. Sharma, H. Adarsha, H. Nagabhushana

**Affiliations:** 1 Department of Chemistry, P. G. Centre, Kuvempu University, Kadur, 577 548 India; 2 Department of Physics, FMPS, M.S. Ramaiah University of Applied Sciences, Bengaluru, 560 054 India; 3 Prof. C.N.R. Rao Centre for Advanced Materials, Tumkur University, Tumkur, 572 103 India; 4 National Assessment and Accreditation Council, Bengaluru, 560 072 India; 5 Jain University, Bengaluru, 562 112 India; 6 Centre for Energy, Indian Institute of Technology, Guwahati, 781 039 India; 7 Department of Mechanical Engineering, Faculty of Engineering and Technology, Jain Global Campus, Bengaluru, 562 112 India

**Keywords:** Nanoscience and technology, Optics and photonics, Physics

## Abstract

Aggregation-induced emission based organic heterocyclic luminogens bearing conjugated electronic structures showed much attention due to its excellent fluorescence in aggregation state. In this communication, a novel conjugated blue light emitting imidazole molecule is synthesized by one pot multicomponent reaction route is reported for the first time. The prepared molecule exhibits a strong fluorescence in aggregation state with exceptional properties, such as high purity, inexpensive, eco-friendly, large scale production, high photostability, etc. By considering these advantages, a new fluorescence based platform has been setup for in-situ visualization of latent fingerprints and its preservation by spray method followed by Poly(vinyl alcohol) masking. A clear and well defined fluorescence fingerprint images are noticed on variety of surfaces by revealing level 1–3 ridge features upon ultraviolet 365 nm light exposure. The dual nature of binding specificity as well as excellent fluorescence properties permits the visualization of latent fingerprints for longer durations (up to 365 days) with superior contrast, high sensitivity, efficiency, selectivity and minimal background hindrance. We further fabricated unclonable invisible security ink for various printing modes on valuable goods for protection against forging. The developed labels are displaying uniform distribution of ink and exceptional stability under various atmospheric environments. The development of long preservative information using aggregation-induced emission based luminogen opens up a new avenue in advanced forensic and data security applications.

## Introduction

Fingerprints (FPs) are considered to be personal identity cards as well as information banks owing to their unique permanent features^1,2^. As a result, they can be utilized in unlock tools, biometric identification and similar authentication purposes. Most of the FPs detectable in crime scene investigation was latent and consequently the visualization has created new avenue in various fields, namely medical diagnostics, forensic investigation, health assessment, etc.^3–5^. Till date, numerous chemical as well as physical methods were attempted for visualization of latent fingerprints (LFPs). However, most of the traditional techniques suffer from several drawbacks, namely low sensitivity, contrast, selectivity, sturdy background hindrance as well as high toxicity^6–10^. Among them, the most commonly used powder dusting technique damages the FPs ridge characteristics at the time of staining and also dust was inevitably dangerous to the developers. Moreover, the chemicals used in these techniques can create a problem to the eyes, skin, DNA or mucous membranes^11–15^. Therefore, developing of LFPs by spraying technique is versatile on various simple and complex surfaces. Further, this method offers various advantages, including simplistic, rapid, superior sensitivity and selectivity, easy-to-store, cost-effective, as well as large-area operable, which is considered to be utmost favourable technique for visualization of LFPs^16^. Generally, in image processing, digital data can be forged or easily ruined. As a result, the safe guarding storage data along with effortless retrieval technique is highly essential. It was the matter of high concern once it involves the FPs protection of extremely wanted criminals^17^. The digitally stored FPs can be effortlessly manipulated by many ways. Till date, several methods have been attempted to secure the digitally stored FPs. For instance, Draper et al.^18^ proposed a simple method to encode fingerprint biometrics securely for long storage, but fails in system security as well as the detailed authentication. In addition, many researchers are seriously attempted on cryptographic aspects of the problem^19–21^. Further, encoded FPs have also stored digitally, which can be subsequently manipulated from these techniques^22^. Hence, physically developed FPs which can be preserved for longer duration was highly essential for crime investigations.

In recent years, counterfeiting of security documents, namely currency, certificates, passport, pharmaceutical products have significantly imbalances the economic condition of the world^23–25^. In addition, the easy accessibility of anti-counterfeiting (AC) inks and its validation for the counterfeiters are the key anxieties in this area. Hence, progress of the AC and authentication methods having multilevel securities was a key aspect to combat counterfeit^26,27^. Till date, stimuli-chromic as well as photoluminescent materials, (quantum dots, carbon dots, polymer nanoparticles, organic and inorganic metal complexes, supramolecular structures, polymer dots, up and down-converting nanomaterials, etc.) are highly suitable for the fabrication of complex AC inks^28–32^. For instance, the carbon dots generally display broad emission spectra and frequently cause overlapping of spectroscopic profiles^33^. Moreover, semiconductor quantum dots prepared using hazardous raw materials can emit narrow emission peaks^34^. In addition, synthesis of metal oxide frameworks is found to be low yield and display excellent luminescent properties with superior compatibility. Hence, fabrication of novel materials are still a challenging task for material scientists to overcome from the existing limitations.

Aggregation-induced emission (AIE) based organic fluorescent materials finds numerous advantages owing to its intensive fluorescence, excellent contrast, simple functionalization procedure^35,36^. In addition, they can also be utilized in imaging and sensing agents. Most of the fluorescent materials suffers severe self-quenching in its aggregation state. From past decades, AIE based materials were utilized to visualize LFPs and AC ink, due to their less toxicity, easy development procedure, superior resolution, etc.^37,38^. On the other hand, some of the AIE based materials also have drawbacks in real-time applications, including large quantity of powder required at the time of staining process. Further, inhalation of the dust particles may be harmful to the developers particularly for smooth substrates^39,40^. Table S1 shows the up to date reported AIE based materials for visualization of LFPs and AC applications^41–49^. The disadvantages of AIE molecules are (i) that commonly used binary organic solvent system which can damage the FPs, (ii) preparation of the developer is worrying because different substrates usually need different solvent ratios, (iii) most of the AIE molecules are unstable and prone to aggregate, which is not conducive to long-term preservation, (iv) they are customarily suitable for smooth substrates, (v) analysis for LFPs on level 3 details (Table S2^8,50–57^) is rarely reported due to the limitation of the imaging mechanism. To overcome from above mentioned barriers, exploring the new AIE based fluorescent materials which are versatile on various surfaces and reveal level 3 details are in urgent need.

To address the above issues, it is essential to fabricate alternate probes for LFPs visualization as well as AC applications by showcase the following characteristics: (i) simple fabrication and development procedure, (ii) non-toxicity, (iii) avoiding aggregation induced quenching, (iv) intensive “on–on” fluorescence mode with high photostability, (v) applicable for porous/semi-porous/non-porous surfaces and (vi) long storage ability of the developed FPs. Herein, we have fabricated an 2-[1-(*9H*-fluoren)-4,5-diphenyl-1*H*-imidazol-zyl]phenol (FDIP) molecule with intense blue emission. The FDIP molecule exhibit exceptional ability on visualization of LFPs with fluorescence “on–on” mode by spraying with an optimized FDIP solution under ultraviolet (UV) 365 nm light illumination. The detailed investigation of LFPs development dynamics with respect to effect of dye concentration, stability of the solution, aging of LFPs, prolonged FPs storage, effect of various environmental conditions and effect of substrates generality are studied. Further, level 3 details of developed FPs films, namely sweat pores, shapes of ridge end, distribution of pores are analysed. In addition, we have also developed AC labels by following simple stamping, writing pen, ink-free relief and intaglio printing techniques that relies on UV 365 nm light to encrypt input AC details on the various substrates. Further, the encrypted input labels are highly stable under different environmental conditions, which endorse the favourable applications in the current AC area.

## Synthesis of 2-[1-(*9H*-fluoren)-4,5-diphenyl-1*H*-imidazol-zyl]phenol (FDIP) molecule

**Figure 1:**
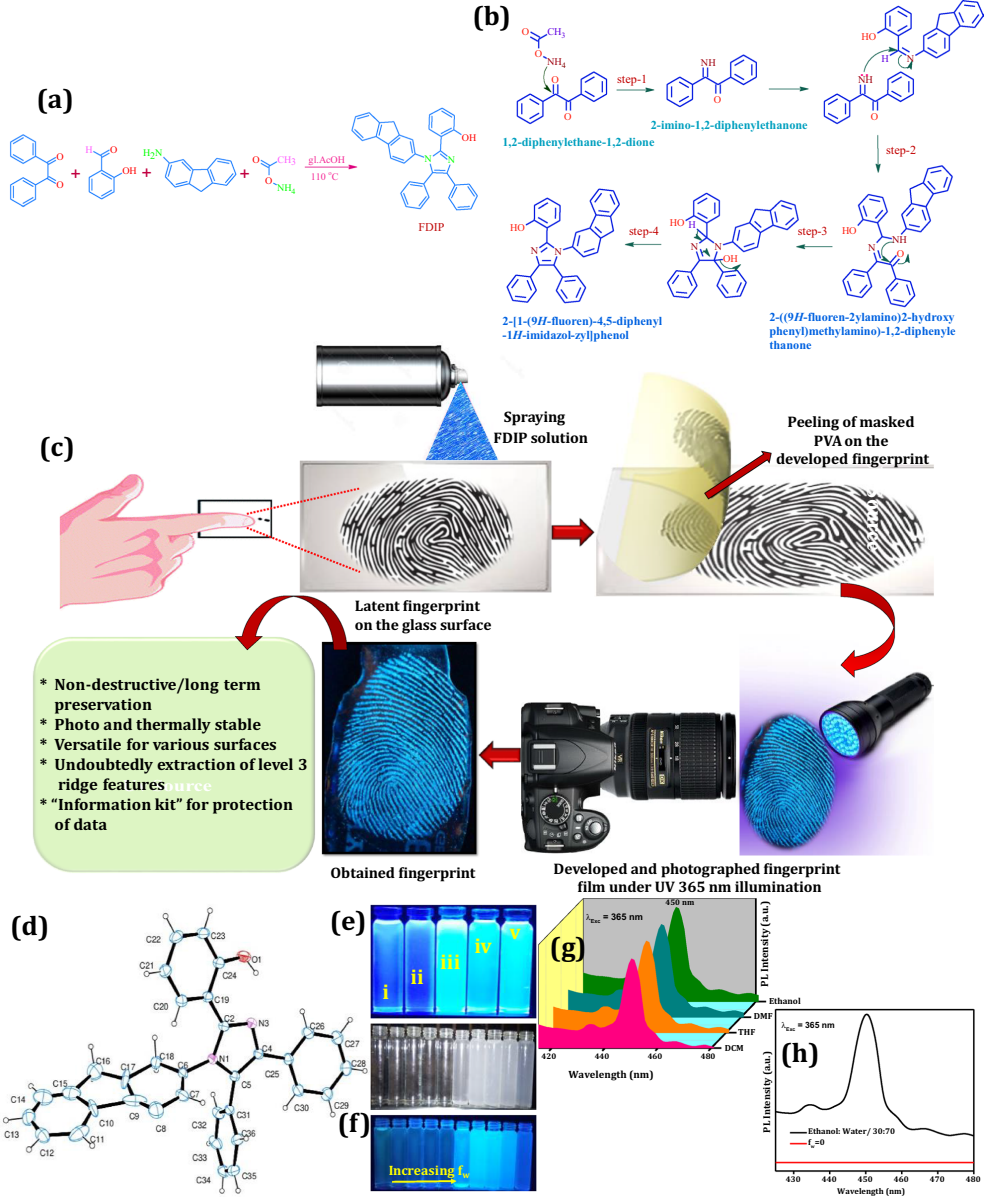
Schematic illustration for the (**a**) synthesis of FDIP molecule; (**b**) step by step chemical reaction involved in synthesis of FDIP molecule; (**c**) development and visualization of long term preservative FPs using optimized FDIP molecule by spraying method followed by PVA wrapping; (**d**) ORTEP diagram of FDIP molecule. Color code: N-violet, O-red, C-blue; (**e**) photographic images of FDIP molecule under UV 365 nm light in various solvents (i) DCM, (ii) DMF, (iii) ethanol and (iv) THF; (**f**) AIE images of the FDIP molecule with various fw values in ethanol under normal and 365 nm light; (**g**) PL spectra of FDIP molecule in various solvents excited at 365 nm light; (**h**) PL spectra of FDIP molecule with fw = 0 and ethanol:water/30:70.

The 1,2-diphenylethane-1,2-dione (1 mmol, 0.210 g), 1-(2-ydroxyphenyl)ethanone (1 mmol, 0.122 g), 9*H*-fluoren-3-amine (1 mmol, 0.182 g), ammonium acetate (1 mmol, 0.229 g) were taken in an equimolar concentration and dissolved in glacial acetic acid (~ 30 ml). The mixture was subsequently ultrasonicated (~ 20 min) and refluxed for ~ 5 h. The chemical reaction was frequently supervised by a thin layer chromatography (TLC) by utilizing appropriate ratio of pet ether and ethyl acetate mixture (7:3). Consequently, the resultant mixture was cool down to room temperature (RT) and transferred to ice water (~ 250 ml). The obtained solution was completely neutralized after the addition of aqueous sodium bicarbonate solution and later on purified through column chromatography by utilizing formerly used solvent chamber ratio to get the desired compound. The schematic illustration for the synthesis of FDIP molecule was depicted in Fig. 1a.

### Appearance

White crystalline solid; Yield: 70–75%; mp: 276–278 °C.

### Fourier transformed infrared (FT-IR) spectrum (KBr, cm^−1^)

1288.17 (C–O), 1420.89 (Aliphatic C–C), 1601.40 (Ar C=C), 1624.44 (C=N), 2916.78 (Aliphatic C–H), 3002.13(Ar C–H), 3427.73 (O–H).

### ^1^H NMR (400 MHz, CDCl_3_, δ_ppm_)

3.79 (s, 2H), 6.77–6.85 (m, 2H, ArH), 6.95–7.01 (m, 2H, ArH), 7.10–7.32 (m, 11H, ArH), 7.54–7.66 (m, 4H, ArH), 8.06–8.14 (m, 2H, ArH).

### Mass

m/z = 477.18 (M^+1^).

## Characterization

The X-ray diffraction data of the prepared FDIP molecule was collected on a Bruker Smart CCD Area Detector System using M_o_K_α_ (0.71073 Å) radiation. The data were reduced using SAINT-Plus. The structure was solved by direct methods using SHELXS97 and refined by difference Fourier synthesis using SHELXL97. The positions and anisotropic displacement parameters of all non-hydrogen atoms were included in the full-matrix least-squares refinement using SHELXL97 and the procedures were carried until convergence was reached. The hydrogen atoms were fixed geometrically and were refined anisotropically. Molecular diagrams were generated using ORTEP. ^1^H-NMR spectra was recorded by using a Jeol-delta with operating frequency of 400 MHz in deuterated chloroform (CDCl_3_) solvent. FTIR spectral data of the prepared samples were recorded using a Nicolet 5700 FT-IR instrument with KBr pellets. Mass spectrometry (LC–MS) was performed with an 1808036-ABS 4 (0.085) Cm (4:7) mass spectrometer. The Jobin Yvon made Fluorolog-3 spectroflourimeter (Xe lamp, 450 W) was used for photoluminescence (PL) studies. High resolution FPs images were captured in a Hitachi (TM-3000) made scanning electron microscope (SEM).

### Development of long term preservative FPs using FDIP

All the fingermarks were collected from the different donors. Firstly, hands were washed thoroughly with soap. The fingers were then gently wiped across the forehead. Finally, the fingers were pressed on the surfaces of different porous, semi-porous and non-porous substrates surfaces at room temperature to obtain LFPs. Subsequently, the optimized aqueous solution of FDIP molecule (Ethanol: Water/30:70) was sprayed on the developed LFPs and air dried. However, the aqueous PVA solution was prepared by dissolving specific amount of PVA (25 g) in a double distilled water. The specific amount ~ 5 ml of PVA solution was wrapped on the developed FPs. Later, the PVA film was peeled slowly and stored in an air tight container to long preservation. The developed FPs films were photographed in situ using a DSLR Canon EOS 100D camera with 5 mm focal point (SIGMA MACRO, 50 mm, F2.8, EXDG) under visible and UV 365 nm light. Figure 1c display the cartoon representation for the development and visualization of long term preservative FPs using optimized FDIP molecule by spraying method followed by Poly(vinyl alcohol) (PVA) wrapping. Abrasion test (physical) was carried out via mounting adhesive tape on the developed FP films and slowly peeled off and photographing under UV light irradiation.

### Fabrication of anti-counterfeiting ink using prepared FDIP

The aqueous solution of FDIP molecule (Ethanol: Water/30:70) was filled in fountain and sketch pen as an ink and written on normal paper surface to demonstrate the AC property of the prepared AIE based ink. Furthermore, the AC patterns were also designed on various surfaces using the optimized ink followed by simple stamping method. The encoded patterns were in situ photographed under normal as well as UV 365 nm light.

## Result and discussions

### Chemistry

The FDIP molecule was prepared via simple acid catalysed to form five membered N-heterocyclic ring. The typical step by step chemical reaction involved in synthesis of FDIP molecule was given below (Fig. 1b);

**Step-1**: Benzil reacts with ammonium acetate which results in 2-imino-1,2-diphenylethanone.
**Step-2**: A lone pair of electron on nitrogen of 2-imino-1,2-diphenylethanone reacts at imine carbon of Schiff base, which formerly obtained by the condensation of salicylaldehyde and 2-aminoflurene to form 2-((9*H*-fluoren-2-ylamino)(2-hydroxyphenyl)methylimino)-1,2-diphenylethanone.
**Step-3**: The 2-((9*H*-fluoren-2-ylamino)(2-hydroxyphenyl)methylimino)-1,2-diphenylethanone undergoes intramolecular cyclization by rearrangement reaction of lone pair electrons of nitrogen (NH group) at electron deficient carbon of carbonyl group to form an intermediate.
**Step-4**: The obtained intermediate undertakes dehydration followed by aromatization to result in final FDIP molecule.

### Spectral data analysis

FTIR spectrum of the prepared FDIP molecule was depicted in Fig. S1. The characteristic bands at ~ 1624 and 1601.40 cm^−1^ were attributed to (C = N) in imidazole ring and aromatic C=C vibrations in benzene ring, respectively. A sharp absorption bands at ~ 3002.13 and 2916.78 cm^−1^ were corresponding to aromatic and aliphatic C–H vibrations. In addition, a broad absorption band at ~ 3427 cm^–1^ corresponding to phenolic –OH group. Figure S2 shows ^1^H NMR spectrum of the prepared FDIP molecule. As evident from the figure, a singlet at ~ 11.74 δ, which confirm the phenolic –OH group. The large δ value of proton may be due to intramolecular hydrogen bonding between oxygen and nitrogen. The six multiplet peaks including 21 protons at ~ 6.38–7.82 δ, assigned to aromatic ring. Further, a singlet peak at 3.98 δ have two protons corresponds to fluorene group. The obtained results clearly evidence the formation of FDIP molecule. The spectral value (m/z = 477.18 (M^+1^)) of the FDIP molecule obtained from mass spectrum was well matched with theoretical value (m/z = 476.56) (Fig. S3). A pure white transparent FDIP crystals was obtained by dissolving in partially soluble ethanol at 50 °C followed by slow evaporation at room temperature for the period of 3 days. The structure refinement parameters and respective hydrogen bond interactions were shown in Table S3. CCDC number-2089983 contains the crystallographic data of FDIP. The ORTEP view with atom labelling (thermal ellipsoids drawn at 50% probability) was shown in Fig. 1d. The target compound crystallizes in monoclinic with space group P2_1_/c. The imidazole and phenol rings were nearly coplanar due to their dihedral angle of 18.72°. The plane containing the fluorene ring and both phenyl rings C25–30 and C31–36 subtends dihedral angles of 75.84, 10.80 and 85.67°, respectively with the central planar imidazole ring. Packing arrangements and photophysical properties of FDIP molecule as described below: The O1 atom of the phenol ring and the N3 atom of the imidazole ring form a short and strong intra-molecular O1–H1···N3 hydrogen bond, forming a S(6) ring, which helps to establish the imidazole and phenol rings close co-planarity displayed in the Figure S4. In the packing diagram of the title molecule, no additional intermolecular, C–H···π and π–π interactions were evidenced. The target crystals emitted strong blue fluorescence under 365 nm light and presented the maximum mission at 450 nm, which were deemed to a reason for the excellent luminous efficiency^58^. Further, based on the spatial distribution of molecular orbitals also the luminescence property of organic molecule can be predicated^59^. The ground state optimized molecular structures; HOMO and LUMO of all compounds were shown in Fig. S5. The FDIP molecule shows large HOMO value (ΔE_HOMO_ =  − 8.063 eV) and small energy band gap (ΔE = 2.860 eV) with respect to LUMO value (ΔE_LUMO_). The least band gap which leads easy to excite the electron from HOMO, it was the essential properties for OLED’s because lowering of the HOMO–LUMO gap was fundamentally a consequence of the large stabilization of the LUMO. The electron clouds of HOMO energy levels were all mainly located on the 4,5-diphenyl-4,5-dihydro-*1H*-midazole of the target molecules and this data strongly counts for good electron donating property and LUMO energy levels were mainly concentrated on the *9H*-fluorene unit, which was useful in the application of OLEDs due to increase in the enhancement of electron transfer^60^.

### AIE feature of FDIP molecule

Figure 1e depicts the photographic images of FDIP molecule dissolved in various solvents, such as dichloromethane (DCM), dimethylformamide (DMF), ethanol as well as tetrahydrofuran (THF) upon normal and UV 365 nm light illumination. It was evident that, FDIP molecule in ethanol shows highest emission intensity upon UV light irradiation as compared to other solvents. This result was further validated by PL studies of the prepared solutions excited at λ_Exc_ = 365 nm (Fig. 1g). The spectrum exhibits a broad peak at ~ 450 nm, owing to the π → π* transition as well as rotation of aromatic ring. The highest intensity was noticed in FDIP in ethanol solvent, and henceforth this was used for further studies. Further, the photographic images of FDIP molecule dissolved in ethanol/water solutions with various water fractions (f_w_) and its corresponding PL emission spectrum was shown in Fig. 1f,h. As evident from the figure, without significant change in PL intensity for lower water proportions up to f_w_ = 40%. The PL intensity reaches its maximum value at f_w_ = 70% and later gradually diminished. The observed variations in the PL intensity was ascribed to following aspects; Firstly, formation of molecular aggregates after expanding the water fraction (poor solvable) there is a diminishing in solubility of FDIP molecule in the ethanol/H_2_O combination because of its inherent hydrophobic nature. This may lead to more intermolecular interactions, which prompts the isolated molecules to involve in the aggregate creation. The aggregation mechanism hindrances the free space accessible for intermolecular rotation and vibration. This confines the rotation of the phenyl rotors and suppresses one of the prevailing non-radiative decay pathways. Subsequently, the excited molecules return to their ground state via fluorescence. Secondly, common solvent impacts the molecular absorption in FDIP molecule was mainly ascribed to the intramolecular charge transfer (ICT) from the ring nitrogen to the carbonyl moiety. Therefore, the supplement of water may enhance the ICT mechanism by balancing the more polar excited state^47^. It was evident that the AIE property depends on the contribution of polar and poor solvation property of water. Conversely, a drastic diminishing nature of emission intensity was observed when f_w_ value is > 70%, which may be due to diminished dissolvability.

### Development and visualization of LFPs films using FDIP solution

**Figure 2:**
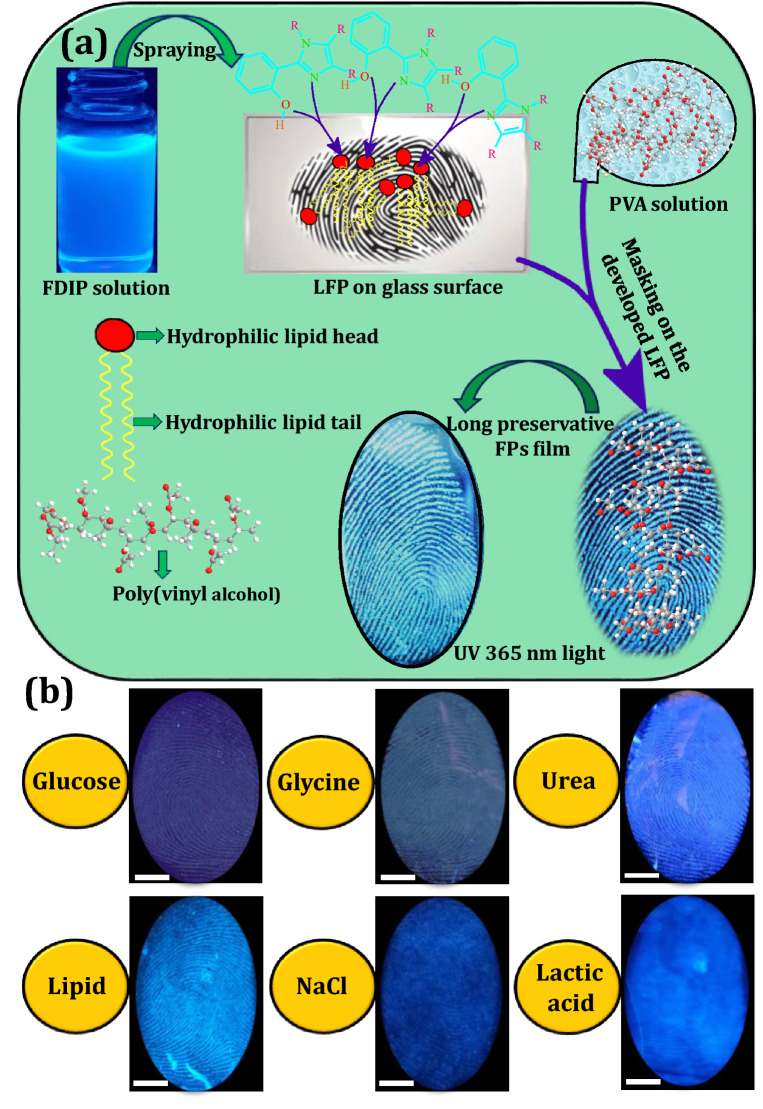
(**a**) Mechanism of LFPs visualization dynamics using FDIP solution; (**b**) RGB true-color FPs images developed on the glass surface soaked with glucose, glycine, urea, sodium chloride, lactic acid as well as lipids solutions under 365 nm light (Scale bar: 5 mm).

**Figure 3:**
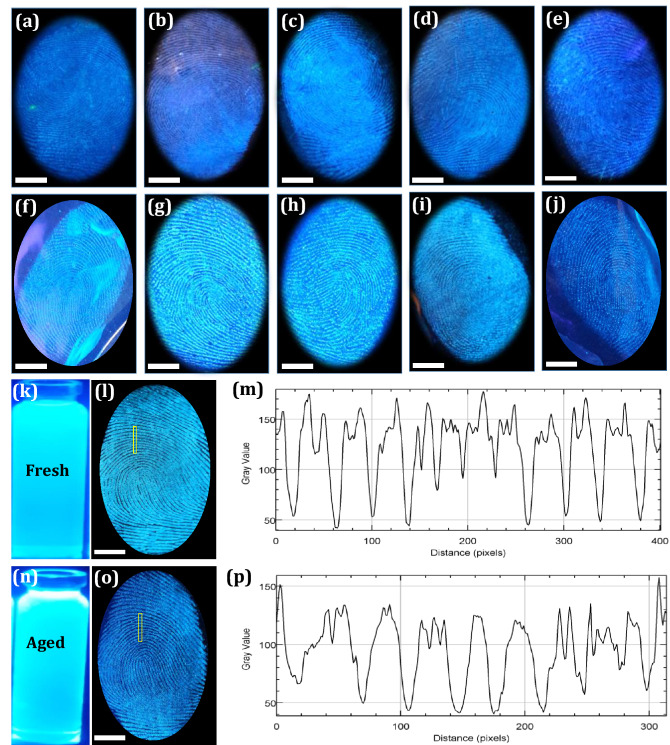
(**a–j**) FPs images developed using various binary mixture concentrations (Ethanol–water fraction) of prepared FDIP molecule on glass surface upon UV 365 nm light illumination; (**k,l,n,o**) Fresh and aged FDIP solution after storing for 3 months and used for LFPs development on glass surface; (**m,p**) Gray scale profiles of the corresponding images of (**l,o**) (Scale bar: 5 mm).

From the past few decades, quantum of work has been attempted to explore the various constituents present in the LFPs. Generally, LFPs comprised with secretory glands in the dermis, epidermis, intrinsic constituents (metabolites and traces of medications and drugs) as well as extrinsic impurities (moisturisers, dirt, cosmetics, blood, grease, hair care products and food contaminants^61^. Further, both intrinsic and extrinsic components considerably vary with individuals (inter-variability) from day to day (intra-variability)^62^. Normally, the intrinsic constituents of the LFPs were comprised of water (95–99%) and organic- inorganic three-dimensional complex emulsions^63^. The eccrine constituents were composed of ~ 98% water, besides inorganic and organic compounds. Till date, more than 20 more amino acids were quantified in the FPs by various techniques. In addition, the sebaceous sweat was composed of many organic constituents, among them common were lipids (glycerides, cholesterol, fatty acids, squalene, lipid esters, sterols). Figure 2a depicts the mechanism of LFPs visualization dynamics using FDIP solution. In order to know the main component of the LFPs, a series of solutions were prepared using glucose, glycine, urea, sodium chloride, lactic acid as well as lipids (Fig. 2b). The previously prepared FDIP solutions were soaked (~ 10 min) on the glass surface and FPs were impressed on it. Later, the FDIP solution was sprayed on the LFPs and visualized under UV 365 nm light. A well-defined FPs ridge patterns were obtained on lipids soaked surface compared to other ones. This may be due to strong interaction between hydrophilic head of the lipids as well as hydrophobic end of FDIP molecule, which results in strong binding between lipids and FDIP molecule (Fig. 2a). The above results clearly revealed that lipids present in the LFPs have greater affinity towards the prepared FDIP molecule. Further, fluorescence imaging was one of the prime tool in LFPs development due to high contrast as well as low background interference. In recent years, AIE based materials were considered to be more efficient to avoid the characteristic aggregation-caused quenching (ACQ) properties compared to conventional fluorescent materials. In addition, AIE based molecules were prepared in a binary system solution (usually acetonitrile/water or ethanol/water) exhibit lipophilicity in nature, hence they can be utilized as an efficient luminescent probes for LFPs visualization. Based on previous reports, the powder dusting and solution method were used for extraction of LFPs using AIE based molecules^44,50^. Among two methods, the solution technique was extensively used owing to its rapid, simple operational procedure and consumption of material was very less. Herein, various binary mixture concentrations (Ethanol–water fraction) of the prepared FDIP molecule for visualization of LFPs films on glass surface upon UV 365 nm light illumination was performed (Fig. 3a–j). As can be observed from the images, an intense and well defined ridge features (type 1–3) were clearly revealed in 7:3 of Ethanol–water fraction. However, the visibility of the developed FPs was also retained in other concentrations, but fail to reveal all types of ridge details. Among the studied results, 7:3 of Ethanol–water fraction was found to be superior than other concentrations. Henceforth, this optimized concentration was used for further detailed investigations. The stability of the optimized FDIP solution was examined by extracting FP films on glass surface with a fresh and aged FDIP solution for 90 days by utilizing spraying technique (Fig. 3k,l,n,o). From the images, it was noticed that indistinguishable FP patterns were obtained by using the formerly prepared solutions without substantial variations in fluorescence signal contrast. In addition, changes in the emission intensity of the FPs furrows and ridges across the yellow box was studied (Fig. 3m,p). Gray scale profiles clearly show the fluorescent signal indicating that the FDIP solution exactly stocked on the ridges than furrows. These results demonstrate the stability of the optimized FDIP solution even for longer duration.

**Figure 4:**
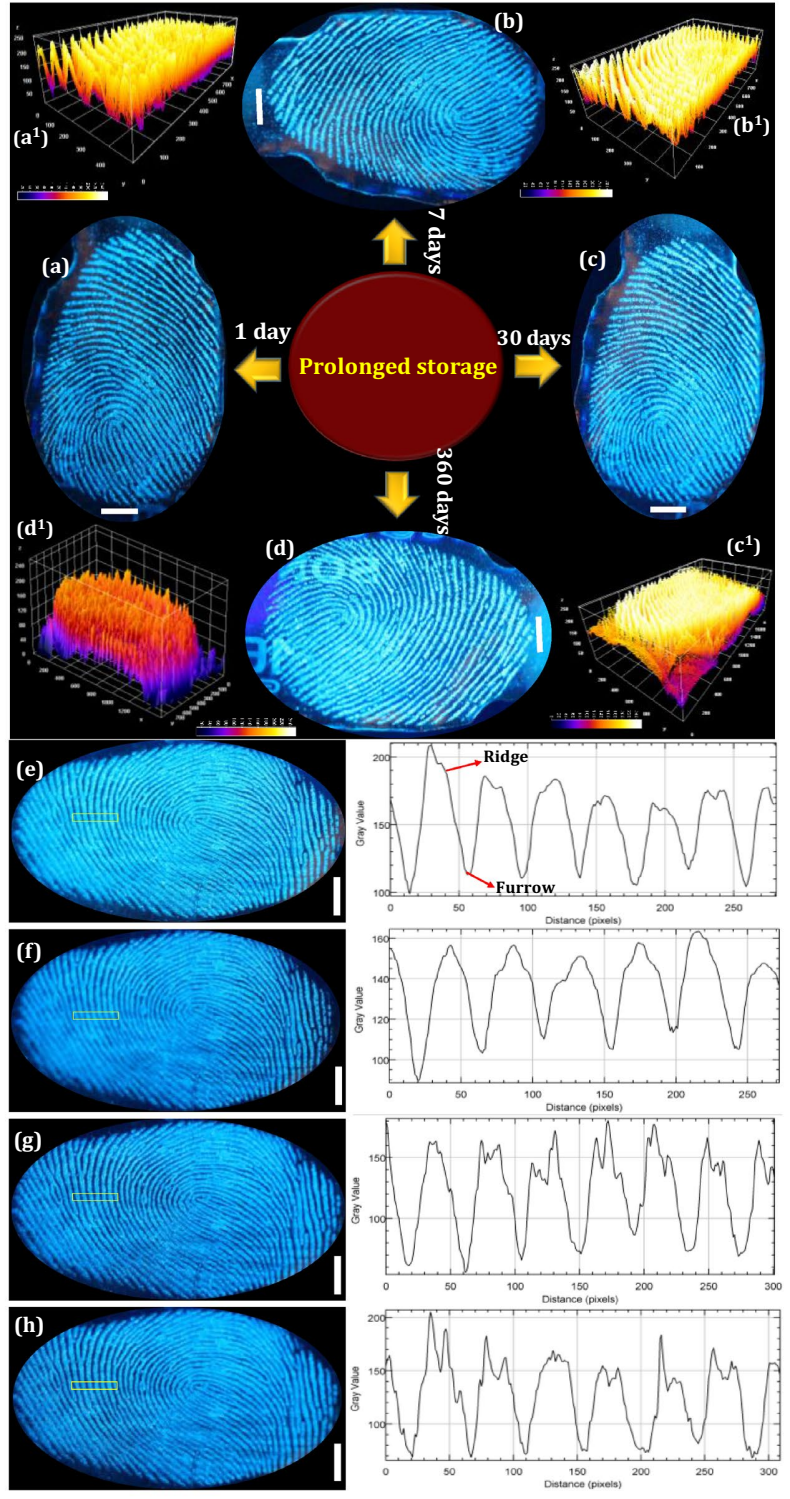
Fluorescence photographs of FP films developed using FDIP solution on glass surface and stored for (**a**) 1 day, (**b**) 7 days, (**c**) 30 days, (**d**) 360 days (The FPs are from the same finger); (**a**^**1**^**–d**^**1**^) 3-dimensional interactive plots of the corresponding FP (**a–d**) (showing uniform distribution of the prepared solution over the ridges); (**e–h**) fluorescence images of donor fingers impressed on the surface of the glass substrate and subsequently aged for 1, 7, 30 and 90 days. The corresponding gray scale profiles of each images of (**e–h**), display fluctuations of the gray values between FP ridge and furrow across the yellow line (Scale bar: 5 mm).

**Figure 5:**
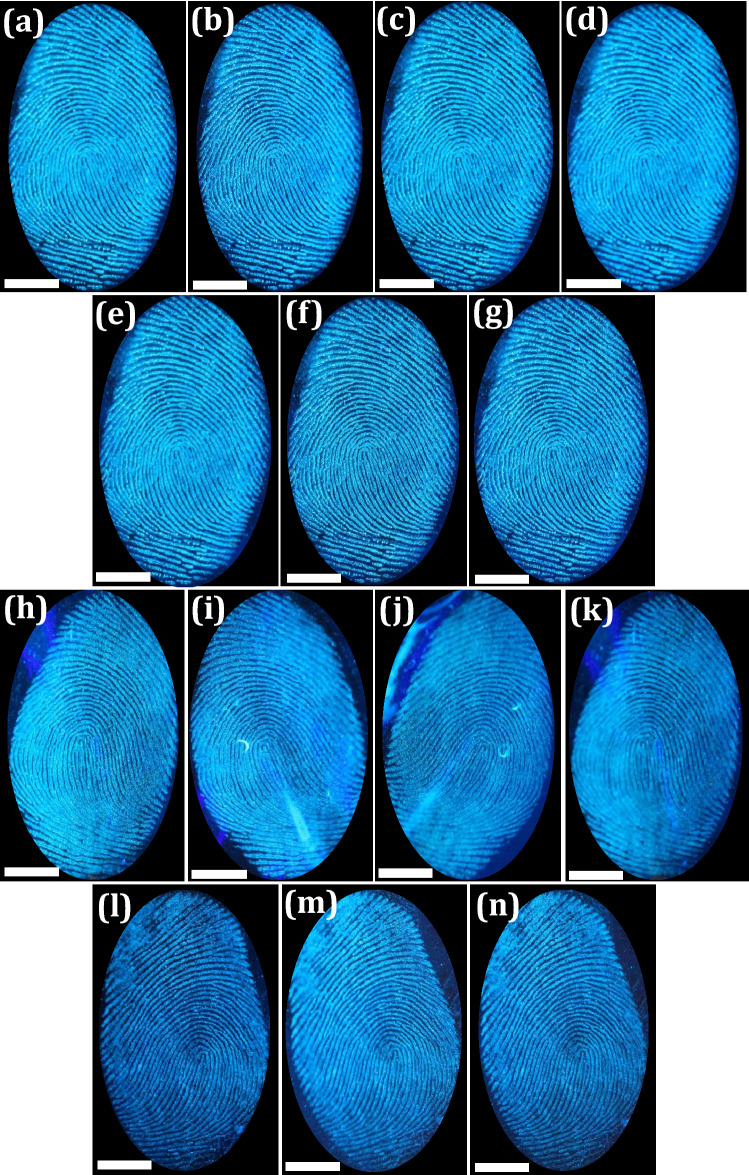
Stability assessment of FP images developed using FDIP solution on the glass surface; (**a–g**) developed FPs was examined upon high intensity UV 365 nm illumination (2 mW/cm^2^) 0 to 6 h; (**h–k**) FPs maintained at different temperatures (30, 50, 100 and 150 °C) for 30 min; (**l–n**) FPs developed under various weather as well as time durations (May, temperature ~ 45 °C, humidity 47%; September, temperature ~ 30 °C, humidity 81%; January, temperature ~ 15 °C, humidity 40%) The corresponding grayscale images were shown in Fig. S6 (Scale bar: 5 mm).

**Figure 6:**
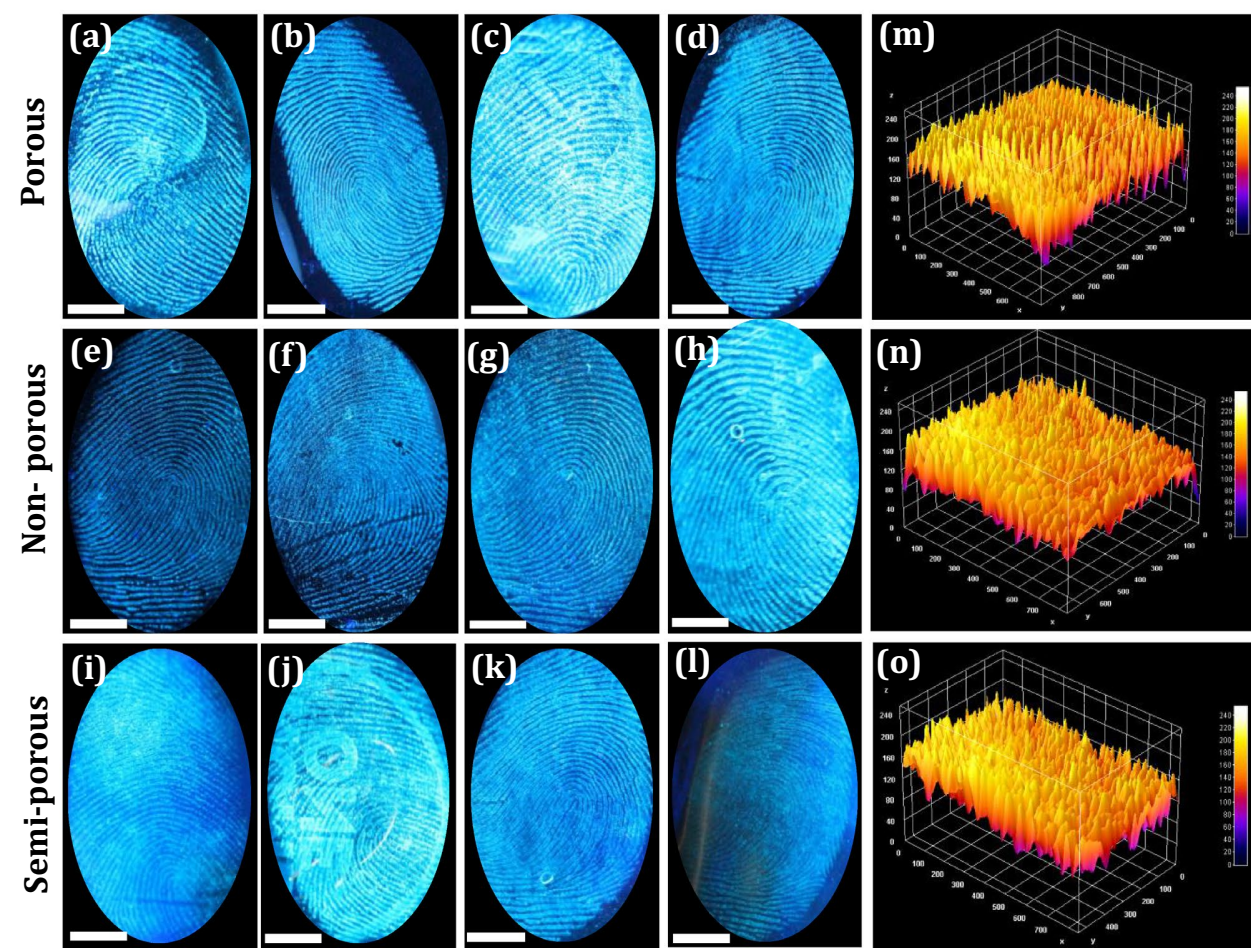
FPs images on different surfaces (**a–d**) non-porous, (**e–h**) porous, and (**i–l**) semi-porous substrates developed by spraying with FDIP solution followed by PVA masking and visualized under UV 365 nm irradiation. The corresponding grayscale images are shown in Fig. S7; (**m–o**) 3-dimensional interactive plots of the corresponding FP (**d,e,k**), which reveals the uniform distribution of the prepared solution over the ridges (Scale bar: 5 mm).

**Figure 7:**
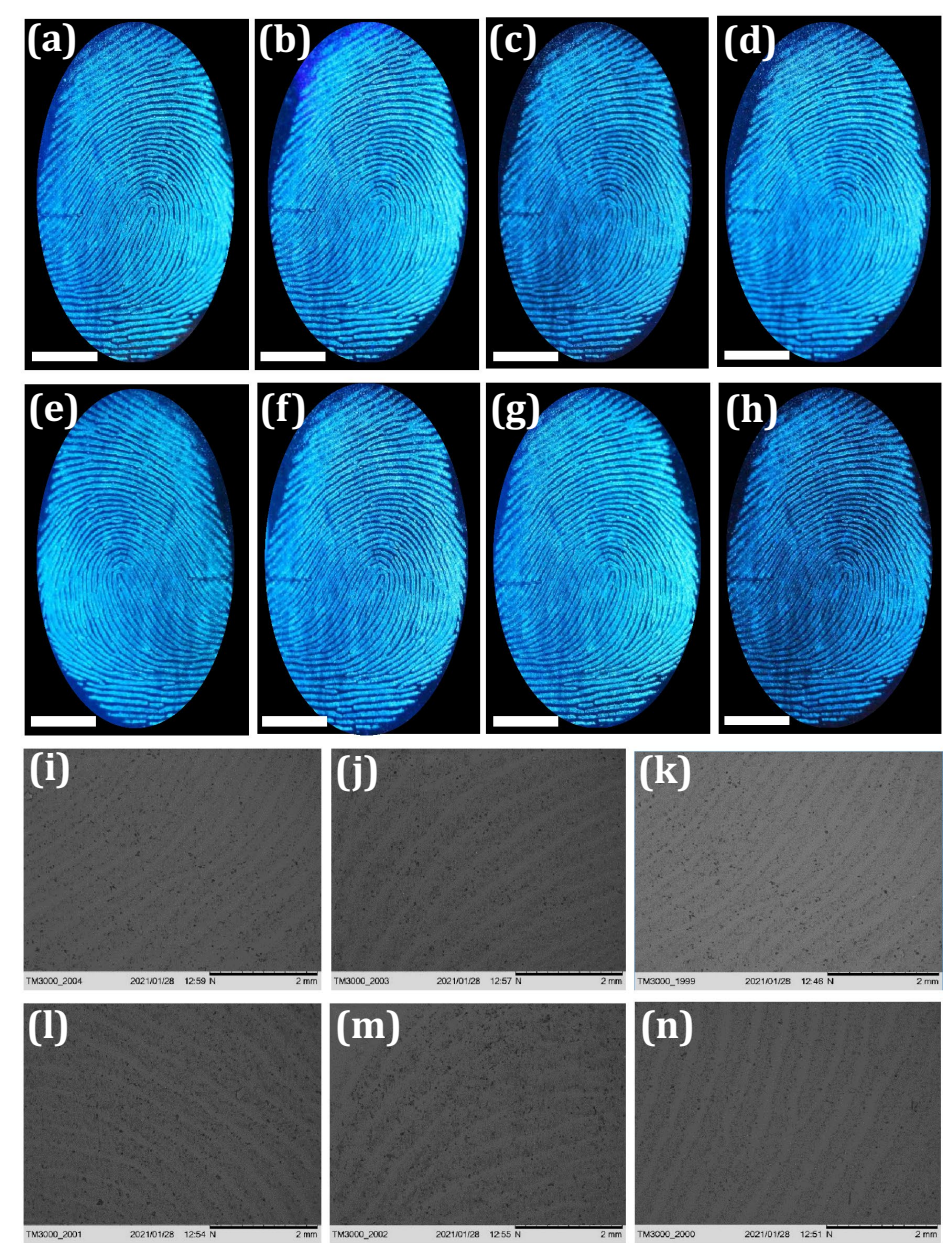
(**a–h**) Fluorescent images of FPs after developing with FDIP solution under UV 365 nm treated with successive physical abrasion. The corresponding grayscale images were shown in Fig. S8 (Scale bar: 5 mm); (**i–n**) SEM images of developed FPs reveal various ridge details demonstrating excellent adhesion of FDIP solution with ridges.

The FPs composition was mostly inconsistent due to substantial variations occur after impression via surface interactions as well as various decomposition and oxidative mechanisms. Normally, FPs compositions can be categorized as (i) initial composition and (ii) the aged composition. These compositions vary via several biological, chemical as well as physical methods ensuing in the aged composition^64^. Hence, detailed investigation was required in order to extract the LFPs mechanisms over various periods. This was broadly categorized into two prospective fields; (i) the LFPs development techniques and (ii) the enhancement of aged FPs. For better understanding of LFPs constituents offers augmented knowledge of the mechanisms and chemical kinetics that can happen between development reagents and their specific FP constituents. This open up a new area of research for advancement in materials and developmental techniques for fresh as well as aged FPs. Furthermore, the conventional reagents, namely Nile red and oil red O were not suitable for development of LFPs aged up to 4 weeks, due to loss of moisture, dispersion of water-soluble components as well as degradation of fragile constituent’s with respect to time^65^. In the present work, we explored the effectiveness of the both developed technique and chemical reagent for the visualization of aged LFPs. The developed FPs films using FDIP solution were stored for ~ 1, 7, 30 and 360 days on glass plate (Fig. 4a–d). As can be seen from the figures, upon UV 365 nm light nearly identical images were obtained with comprising of all the ridge features. The PVA coating improves the physical adhesiveness of the FDIP molecule on various substrates owing to its intrinsic amphiphilicity nature of PVA. Figure 4a^1^–d^1^ depicts 3-dimensional interactive plots of the corresponding FPs. The obtained results clearly authenticate the uniform distribution of the FDIP over the FPs ridges. Furthermore, the donor’s fingers were impressed on the surface of the glass substrate and subsequently aged for ~ 1, 7, 30 and 90 days (Fig. 4e–h). Later, the aged FPs were visualized by spraying FDIP solution under UV 365 nm illumination. The obtained results clearly demonstrated that, the visualization sensitivity slowly diminished with prolonged time. However, FPs aged for up to 90 days could be able to reveal characteristic features (including level 1–3). The corresponding gray scale profiles of each FP images display distinguishable fluctuations of the gray values between FP ridges and furrows. The above results clearly authenticate the prolonged durability of the developed method and prepared FDIP solution for LFPs visualization. The FPs constituents varies after impression and was affected by donor factors, transfer conditions and environmental factors (light exposure, atmospheric pollution, air circulation, dust, friction, humidity, temperature, etc.)^66^. Most of the FPs on surfaces of the substrates available at the crime spot were undergone several environmental conditions, which intern affect the detection sensitivity. Among the mentioned conditions, light exposure was found to affect much on visibility of FPs. For instance, various findings authenticated that the decomposition of squalene happens quickly upon UV irradiation. Jones et al.^67^ studied that the variations of lipid components over the period under various environments and also fatty acids were quickly vanished in dark environments. Herein, the photo stability of FDIP solution for visualization of LFPs on glass surface was examined upon prolonged UV 365 nm illumination up to ~ 6 h (Fig. 5a–g). It was noticed that, no obvious fluorescence quenching. Further, the prepared solution was utilized to visualize the LFPs on the glass surface followed by spray method under UV 365 nm light. The obtained results clearly showed well defined ridge details, indicating that UV exposure will not influence much on the visualization ability and its corresponding grayscale images were shown in Fig. S6. The present results validate the outstanding photo stability of the prepared solution. Generally, the rate of water loss in LFPs was more prominent with respect to temperature and humid environment. The high temperature atmosphere of LFPs results in more degradation of amino acids as compared those at RT. Sampson et al.^68^ investigated the optimum temperature (~ 20–35 °C) to develop successful LFPs by utilizing amino acid reagents. The LFPs heat treated for longer time period, visualization would be considerably more challenging, owing to rapid degradation of amino acid. However, acid salts were significantly resistant for higher temperatures and LFPs were still visible, even after heating at ~ 70 °C for 72 h. To validate the sensitivity of the prepared FPs film, a series of experiments were performed by maintaining different temperatures (30, 50, 100 and 150 °C) and maintained for 30 min (Fig. 5h–k). The obtained results clearly indicate that the developed FPs films were highly thermally stable. Besides, variation in the emission intensity between the FP ridges and furrows which exhibit an excellent contrast. It was worth mentioning that the obtained LFPs details clearly withstand even for higher temperatures for several hours. The effect of humidity on LFPs development has been attempted under different weather conditions as well as time durations (May, temperature ~ 45 °C, humidity 47%; September, temperature ~ 30 °C, humidity 81%; January, temperature ~ 15 °C, humidity 40%) under UV 365 nm light (Fig. 5l–n). It was presided that no noticeable variations in the clarity of images, which enabling defined level 1–3 minutiae ridge information. To study the versatility of the developed FPs films on different porous (paper with different background, magazine covers), non-porous (glass, stainless steel, plastic and ceramics) and semi-porous surfaces (leather, cardboard, wood and filter paper) under UV 365 nm irradiation (Fig. 6a–l and the corresponding grayscale images were shown in Fig. S7). High contrast and resolution FPs images were clearly noticed. The visualized FPs on different surfaces clearly exhibit level 1–3 details with high sensitivity and selectivity. In addition, 3D interactive plots were also plotted (Fig. 6m–o). As evident from the figure, the prepared FDIP solution staked exactly on the FPs ridges, whereas, no signature of fluorescence in furrows regions. The clear fluorescence on the ridges even after drying indicating that the sprayed FDIP solution was not endured any outward capillary flow from the center of the liquid towards the edge, showing the suppression of well-known “coffee-ring effect”. Furthermore, a series of experiments were performed on FPs films with up to 7 cycles of physical abrasion and the grayscale images were shown in Fig. S8. Further, the photographic images captured under UV 365 nm light irradiation were also shown in Fig. 7a–h. As evident from the figures, a well-defined FPs images with all ridge features were clearly visualized even after several times of physical abrasion (7 cycles). Hence, the present developmental technique was more convenient in storing FPs films for longer durations. In order to reveal the enlarged ridge features, SEM images were taken from the developed FPs and shown in Fig. 7i–n. A clear distribution of FDIP solution over the ridges and reveals the level 2 details, which enabling to lawful and trustworthy evidence for individualization.

**Figure 8:**
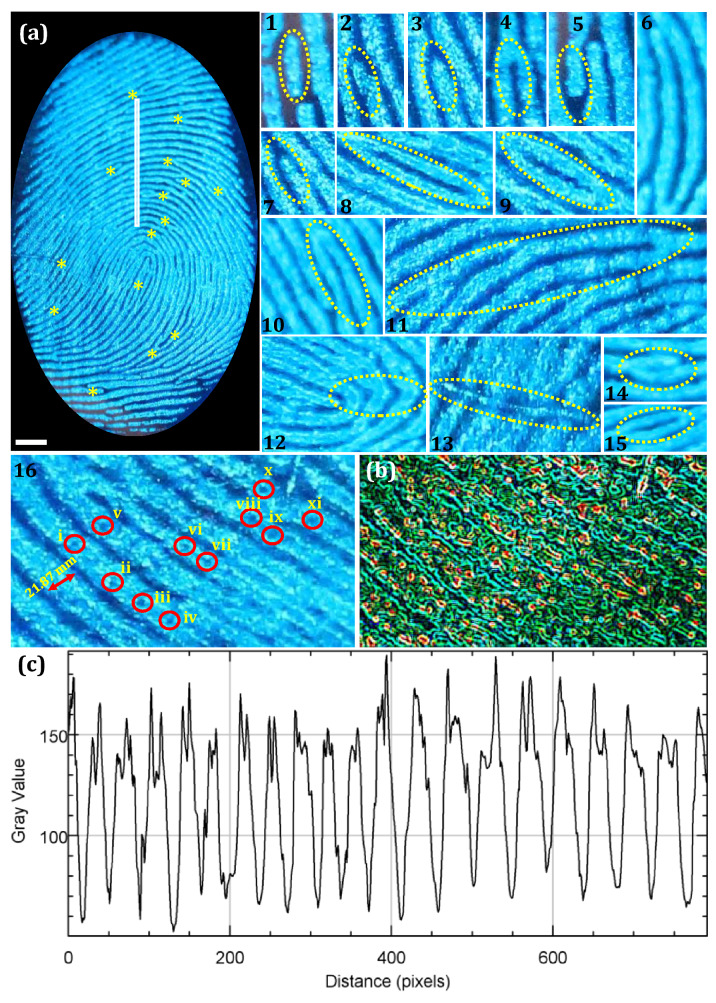
(**a**) High resolution FP image along with extracted ridge features including level I, level II and level III (1–16) on the glass surface visualized by FDIP solution followed by spray method under UV 365 nm illumination. The corresponding grayscale images were shown in Fig. S9; (**b**) interactive plot of the portion of the FP to show the distribution of sweat pores on the ridges of the FP; (**c**) fluctuations in the gray value over the FP indicated by the white bar in the (**a**) (Scale bar: 5 mm).

Normally, FPs were distinguished by three levels of information to personal individualization. Level 1 features were not distinctive enough to personal identification, only denotes the shape, center point and triangle points of the FPs. Further, the level 1 features were classified as whorl, loop, arch, etc. The macroscopic Level 2 information, namely bifurcation, lake, cross-over, bridges, island, short ridge, double bifurcation, etc. Whereas, the level 3 information were the microscopic ridge details of the FP, namely sweat pores, shape of the ridge edge and the width of the ridges^69^. In general, level 1–2 details of FPs were usually available at the crime scene, which were highly difficult to individualize the person. Hence, microscopic level 3 information, namely edge shape, width and narrowness of the ridges and sweat pores become particularly important due to their unique features^70^. Figure 8a shows the images of level 2–3 information developed on glass surface by using FDIP solution under UV 365 nm light (The corresponding grayscale images were shown in Fig. S9). It was noticed that, the high magnified fluorescence images of ridge features, namely whorl, ridge end, bifurcation, ridge termination, hook, scar, trifurcation, lake and short ridge were clearly distinguishable. Further from the images, the number, position, width of the ridges as well as distribution of characteristic sweat pores on the FPs were effortlessly distinguished. The 3D interactive plot of the portion of the FP clearly show the distribution of sweat pores on the ridges of the FP (yellow markings) (Fig. 8b). Furthermore, the fluctuations in the gray value over the FP evidences that the uniform distribution of FDIP solution on the FP ridges (Fig. 8c). Herein, AIE based FDIP solution clearly demonstrates the significant competence on long preservative FPs films through “on–on” fluorescence mode by following spraying method upon UV 365 nm illumination. The present FDIP solution was considered to be excellent fluorescent probe for LFPs development and analysis as compared with previous literature, with respect to; (i) simple preparation method followed by four-step reactions process, (ii) easy to develop preservative FPs films via simply spraying on the LFPs surfaces, (iii) versatile for many surfaces, namely non-filtrating substrates, (iv) high contrast, superior sensitivity and low background hindrance due to the “on–on” characteristic fluorescent probe and (v) reveal of unprecedented microscopic details of such as sweat pores, shape of the ridge edge and the width of the ridges. Herein, we believe that the “on–on” characteristic fluorescent probe can excel effortlessly in the long preservative FPs development procedure, providing an alternate platform for the forensic scientists.

### Application to anti-counterfeiting labels

**Figure 9:**
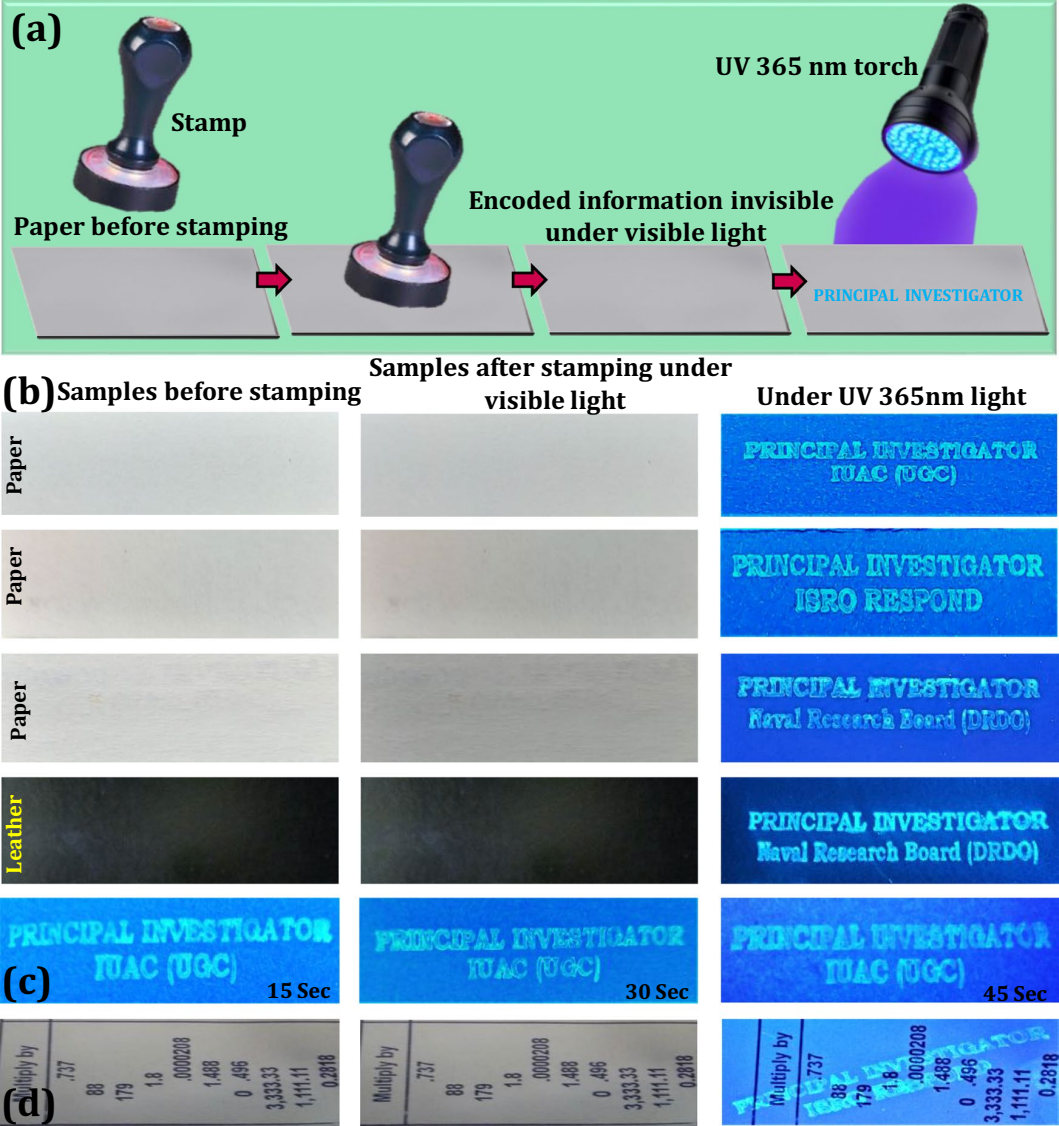
(**a**) Schematic illustration of different input anti-counterfeiting patterns developed on paper surface by following stamping method. UV 365 nm light was used as an excitation source to decode the patterns. The developed information was in situ photographed using a DSLR Canon EOS 100D camera; (**b**) photographs of the word “PRINCIPAL INVESTIGATOR IUAC (UGC)”, “PRINCIPAL INVESTIGATOR ISRO RESPOND” and “PRINCIPAL INVESTIGATOR Naval Research Board (DRDO)” stamped by using FDIP solution on paper and leather surfaces. Left panels were plane surfaces without any stamping under normal light. Middle panels of each photographs exhibit images in visible light after stamping. Right panels were corresponding photographic images of input patterns under UV 365 nm light; (**c**) fluorescence images on paper developed with various stamping time under UV 365 nm light irradiation; (**d**) images of anti-counterfeiting water-mark were developed using FDIP solution on the complex paper surface by stamped method.

**Figure 10:**
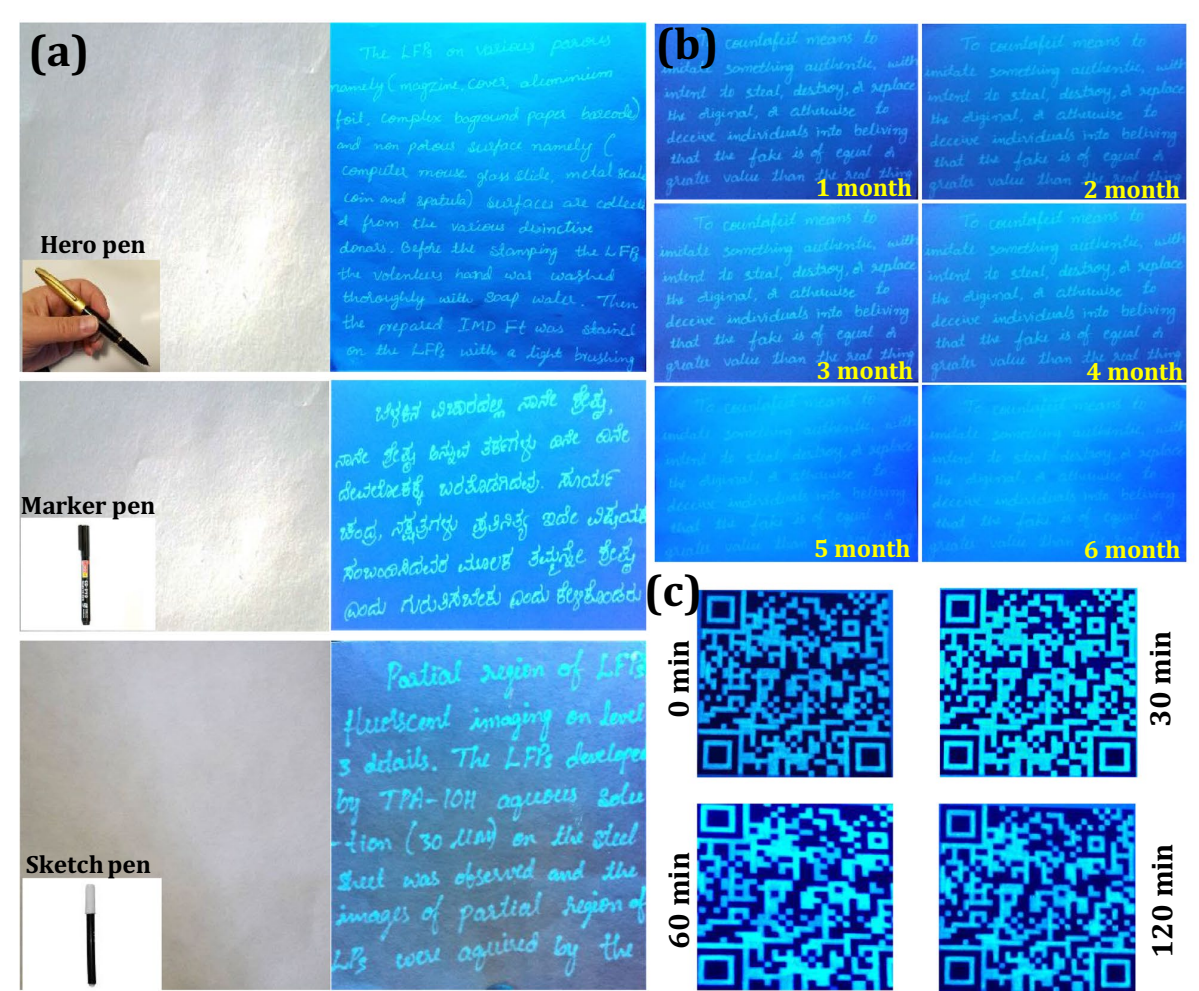
(**a**) Photographic images of English and Kannada words handwritten by hero pen, marker pen and sketch pen under visible and UV 365 nm light excitation; (**b**) fluorescent images of decoded anti-counterfeiting information stored in different time periods at RT; (**c**) photostability of developed QR code information treated with different UV irradiation time periods.

**Figure 11:**
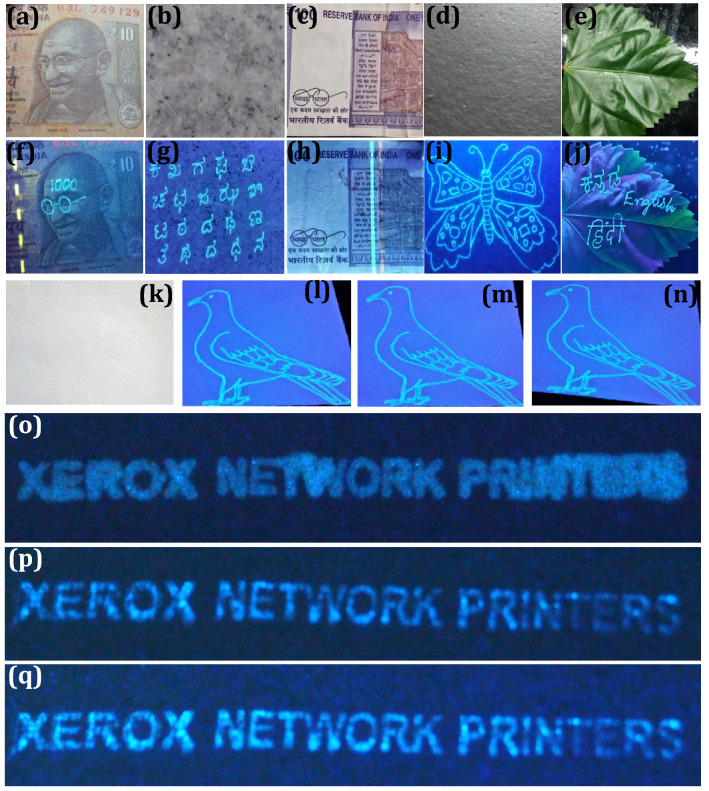
Images of encoded information on various surfaces under (**a–e**) normal light, (**f–j**) UV 365 nm light excitation; (**k–n**) Photographic images of encoded handwritten information on the glassy ceramic tile surface and treated with various time period and temperature (30 days at RT, below 90 °C for 24 h, 100 °C for 30 min); fluorescent images of decoded patterns under UV 365 nm light excitation showing high reliability on the paper surface by treated with (**o**) water, (**p**) oil and (**q**) acetone for ~ 12 h.

Exploring real-time applications of the novel materials like FDIP molecule was always significant, due to its exceptional properties, such as high and steady fluorescence emission as well as superior transparency in the visible region, which makes them appropriate candidates for fluorescent ink applications. The characteristic fluorescent “on–on” probe of the optimized FDIP solution made us to attempt anti-counterfeiting labels by following simple stamping method. Figure 9a shows schematic illustration of different input anti-counterfeiting patterns developed on paper surface by following stamping method. The photographs of the word “PRINCIPAL INVESTIGATOR IUAC (UGC)”, “PRINCIPAL INVESTIGATOR ISRO RESPOND” and “PRINCIPAL INVESTIGATOR Naval Research Board (DRDO)” were stamped on paper and leather surfaces by using FDIP solution (Fig. 9b). It was evident that, the stamped labels did not notice on these surfaces under normal light. However, clear images with uniform distribution of the ink was noticed on the encoded patterns upon UV 365 nm irradiation. In addition, a series of experiments were performed by varying stamping time (15, 30 and 45 s) on the paper surfaces (Fig. 9c). It was clearly demonstrated that the emission intensity of the encoded patterns upon 365 nm excitation can be easily engineered by varying the stamping time. Besides, confidential anti-counterfeiting water-mark were also developed using optimized FDIP solution on the complex paper surface by stamped method (Fig. 9d). Under normal light, there was no evidence of encoded water-mark on the paper, although highly intensive bright encoded water-mark “PRINCIPAL INVESTIGATOR, ISRO RESPOND” on the complex paper surface was clearly noticed upon UV 365 nm irradiation. The obtained results clearly demonstrated that the developed stamping method was quite simple and can be used for protecting the confidentiality of the documents. Further, anti-counterfeiting labels were developed via a hero, marker and sketch pen mode upon loading with optimized FDIP solution and used for handwritten of English and Kannada characters on the normal writing paper (Fig. 10a). As can be seen from the figures that the texted information was undoubtedly visible under UV 365 nm light, however, under normal light the clear blank paper was observed. Besides, the uniform distribution and emission intensity of the texted information on the normal paper was still reliable even after lengthened writings and can be retained even after 6 months when stored at RT (Fig. 10b). The photostability of the encoded information using FDIP solution upon UV 365 nm light irradiation was also studied (Fig. 10c). The developed QR code information was placed at different UV irradiation time period (0, 30, 60 and 120 min) and their corresponding images were photographed. The obtained photographs clearly found that the fluorescence property gradually diminished and became ambiguous above a certain time. This was mainly due to the un-decorated portion exhibit intensive fluorescence upon UV light exposure, although intensity of the decorated QR code reached its maximum value. When the un-decorated portion attains its higher fluorescence intensity like QR code, the difference between the QR code as well as un-decorated portion will not be distinguishable. The obtained results clearly demonstrated that the prepared FDIP solution based counterfeiting labels were used for UV-light sensitive smart labels. Photographic images of encoded information on various surfaces, namely currency, marble, cardboard and leaf under normal light and UV 365 nm light excitation (Fig. 11a–j). It was evident that the encoded information was clearly decoded under UV light, however, it is invisible under normal light. This indicates that the developed AC ink quite useful for most of the industrial applications to combat counterfeiting of the products. The effect of time period and temperature on the encoded pattern was exclusively studied, as shown in Fig. 11k–n. The developed patterns on the glassy ceramic tile can withstand its fluorescence up to 30 days at RT. When the patterns were treated with below 90 °C for 24 h showed any drastic alterations of the fluorescent patterns. Normally, the fluorescent patterns were obliterating under different conditions. In the present work, the developed patterns were heat treated at ~ 100 °C for 30 min results in complete encoded information was erased. Figure 11o–q depicts the reliability of encoded patterns on the paper surface by treated with water, oil and acetone for ~ 12 h. The encoded fluorescence patterns were highly stable without much difference, these outstanding advantages offers the prepared FDIP solution based labels to combat counterfeiting in packaging, food and pharmaceutical industries. These results highlighted the potentiality of the material for smart package material for temperature-sensitive goods.

### Mechanistic study and ink-free printing

**Figure 12:**
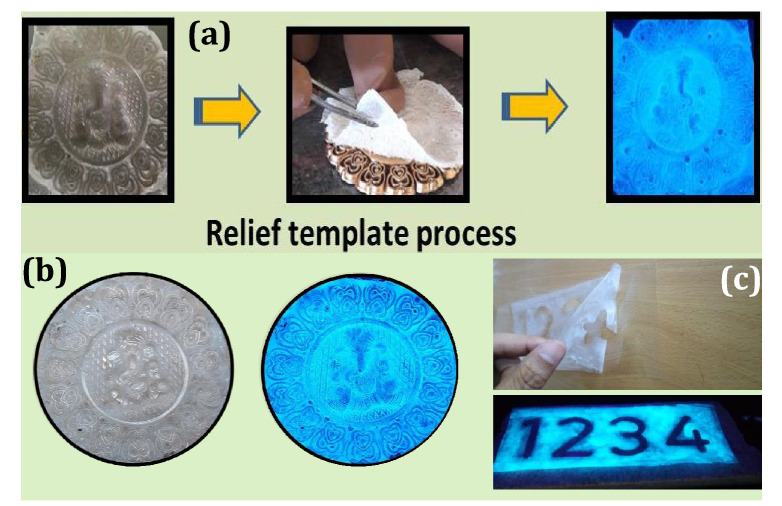
(**a**) Schematic representation of simple ink-free relief template method; (**b**) Photographed images of developed “lord Ganesha” film by relief template method upon visible and UV 365 nm irradiation; (**c**) xylographic images developed by following ink-free intaglio printing method with prepared PVA/FDIP under normal and UV 365 nm light.

The relief template technique was employed to develop anti-counterfeiting labels by simple procedure as follows; a commercially purchased PVA (~ 50 ml) and FDIP molecule (~ 250 mg) were taken in a beaker and irradiated by probe sonicator for ~ 1 h by maintained at a frequency of 22 kHz to obtain a clear and transparent solution. The used PVA medium offers excellent dispersion for FDIP molecule without any clusters which resulting superior viscous nature by providing more adhesive property on the various surfaces of the substrates. Later, the resulted mixture was drop-casted onto a surface of the pre-designed shapes, followed by the evaporation of solvents, which results a flexible transparent film. Then the film was peeled slowly from the surface, and pre-designed shapes was exhibited on the film. This developed film was photographed and analysed upon UV 365 nm irradiation. Figure 12a depicts the schematic representation of simple ink-free relief template method. The relief printing procedure for “lord Ganesha” by using previously prepared mixture as ink-free patterned substrates were shown in Fig. 12b. The obtained result clearly displays fluorescent Ganesha pattern on the film, where intensive fluorescence was noticed in the edge areas of the pattern. Figure 12c depicts the process underlying in intaglio printing by using prepared mixture as ink-free patterned substrates. The prepared mixture was poured on the intaglio xylograph, followed by solvent evaporation. Then film was peeled slowly from the surface resulting in a flexible transparent film. The obtained film comprises with well-developed “numericals” profiles upon UV 365 nm excitation.

## Conclusions

In conclusion, an AIE-based conjugated imidazole molecule was synthesized via one pot multicomponent reaction route. The prepared molecule exhibits intense blue emission by overwhelming aggregation induced self-quenching. Thanks to the “on–on” property of FDIP molecule, in situ fluorescence visualization of LFPs and invisible security ink was effectively executed. The visualized LFPs images were developed by spraying method followed by PVA masking, which was advantageous for long term preservation with high resolution. The developed FPs reveal level 1–3 ridge features, which were reliable evidences for personal identity. Besides, nanoscopic level-3 ridge information, including ridge end shape, sweat pores distribution and ridge width can be revealed in SEM images. The masked films comprised with FPs acting as a new “information kit” used for protection of data and its effortless retrieval. In addition, invisible AC labels were developed by both with and without ink-free techniques. Emission intensity of the encoded patterns upon 365 nm excitation can be easily engineered by varying the stamping time. The encoded AC labels were easily readable upon UV 365 nm light illumination and stable under various conditions. The developed AC techniques were quite useful to combating counterfeiting in various fields.

### Ethical approval

The authors confirmed that all experiments (taking fingerprints of a volunteer/individual) were performed in accordance with relevant guidelines and regulations. An explicit informed consent was obtained from the anonymous volunteer providing the fingerprints. The individual explicitly allowed the authors to use the data in the present publication. And also authors confirmed that all human experimental protocols were approved by a *Tumkur University* institutional committee.

## Supplementary Information

Supplementary Information.
