# Supplementary material for: Aggregation induced emission based active conjugated imidazole luminogens for visualization of latent fingerprints and multiple anticounterfeiting applications
Source: Sci Rep. 2021 Aug 18;11:16748. doi: 10.1038/s41598-021-96011-5 (PMC8373972; doi:10.1038/s41598-021-96011-5)
Supplement: Supplementary file 1 — Supplementary Information. [file 41598_2021_96011_MOESM1_ESM.docx]

**Additional Information**

**Aggregation Induced Emission based Active Conjugated Imidazole Luminogens for Visualization of Latent Fingerprints and Multiple Anticounterfeiting Applications**

**M.K. Ravindra^1^, G.P. Darshan^2^, D.R. Lavanya^3^, K.M. Mahadevan^1^, H.B. Premkumar^2^, S.C. Sharma^4,5,6^, H. Adarsha^7^, H. Nagabhushana^3, *^**

*^1^Department of Chemistry, Kuvempu University, P. G. Centre, Kadur 577 548, India*

*^2^Department of Physics, FMPS, MS Ramaiah University of Applied Sciences, Bengaluru 560 054, India*

*^3^Prof. C.N.R. Rao Centre for Advanced Materials, Tumkur University,*

*Tumkur 572 103, India*

*^4^National Assessment and Accreditation Council, Bengaluru 560 072, India*

*^5^Honarory Professor, Jain University, Bengaluru 562 112, India*

*^6^Distinguished Professor, Centre for Energy, Indian lnstitute of Technology, Guwahati 781 039, India*

*^7^Department of Mechanical Engineering, Faculty of Engineering and Technology, Jain Global Campus, Bengaluru 562 112, India*

Figure S1. FTIR spectrum of the FDIP molecule.

Figure S2. ^1^H NMR spectrum of the prepared FDIP molecule.

Figure S3. Mass spectrum of FDIP molecule.


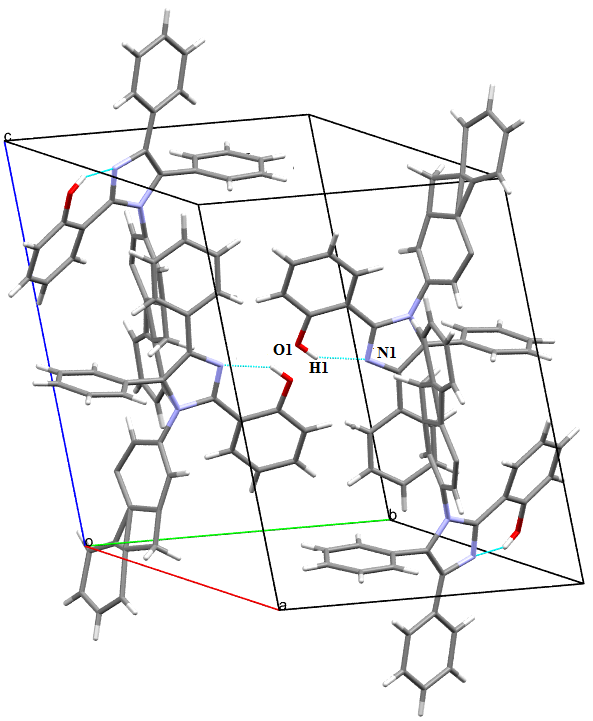


Figure S4. A unit cell packing of FDIP, showing intra-molecular O-H···N and interactions with dotted lines.


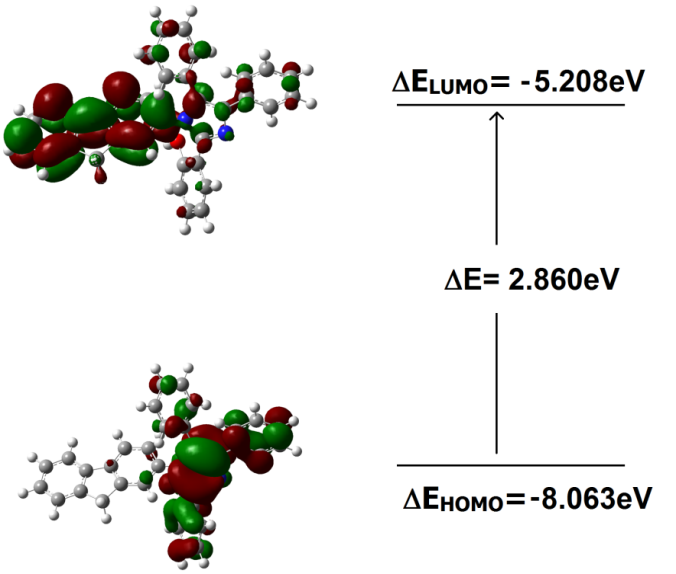


Figure S5. Energy levels, band gap and corresponding frontier orbitals (HOMO and LUMO) of FDIP molecule.

Figure S6. Grayscale images of LFPs developed with using FDIP solution on the glass surface; (a-g) developed FPs was examined upon high intensity UV 365 nm illumination; (h-k) FPs maintained at different temperatures for 30 min; (l-n) FPs developed under various weather as well as time durations (Scale bar: 5 mm). The grayscale images are corresponding to the RGB true color images of Figure 5. Gray value: 0-255.

Figure S7. Grayscale FPs images on non-porous, porous and semi-porous substrates developed by spraying with FDIP solution (Scale bar: 5 mm). The grayscale images are corresponding to the RGB true color images of Figure 6 (a-l). Gray value: 0-255.

Figure S8. Grayscale images of FPs after developing with FDIP solution treated with successive physical abrasion (Scale bar: 5 mm). The grayscale images are corresponding to the RGB true color images of Figure 7 (a-h). Gray value: 0-255.

Figure S9. Grayscale images of high resolution FP image along with extracted ridge features including level I, level II and level III (1-16) on the glass surface visualized by FDIP solution (Scale bar: 5 mm). The grayscale images are corresponding to the RGB true color images of Figure 8 (a). Gray value: 0-255.

Table S1. The reported AIE based materials used for visualization of LFPs.

| **Compounds** | **λ_emi_**  **_(nm)_** | **Light irradiation**  **(nm)** | **Conc.** | **Solvent combination** | **Water fraction** | **References** |
| --- | --- | --- | --- | --- | --- | --- |
| Tetraphenylethene-based Dye (**FLA-1)** | 470 | 365 | 0.25 mM | CH_3_CN/H_2_O | 60 %,  70 % | [41] |
| 9-(4-(ter-butyldiphenylsilyloxy)phenyl)3,3,6,6,10-pentamethyl-3,4,6,7- tetrahydroacridine1,8(*2H,5H,9H,10H*)-dione) **(ADDSi)** | 440 | 365 | 0.3 mM | THF/H_2_O | 60 % | [42] |
| 5-[-(2-hydroxybenzylidene)amino]-2,3-diphenylpyrimidin-4(3*H*)-one (**DPSA**) | 564 | 365 | 1 mM | CH_3_CN/H_2_O | 90 % | [43] |
| 2-(4-(4-(diphenylamino)styryl)pyridin-1(*2H*)-yl)ethanol **(TPA-1OH)** | 658 | 405 | 30 μM | EA/H_2_O | 99 % | [44] |
| 3,3′-((2-(4-(1,2-diphenyl-2-(p-tolyl)vinyl)phenyl)-7-(7-methylbenzo[c][1,2,5]thiadiazol-4-yl)-*9H*-fluorene-9,9-  diyl)bis(hexane-6,1-diyl))bis(1-methyl-*1H*-imidazol-3-ium) bromide (**PFTPEBT-MI).** | 555 and 565 | 365 | 33 μM | THF/H_2_O | 90 % | [45] |
| Carbazole linked anthranyl phosphonate **(CBZP)** | 526 | 365 | 1x10^-5^ M | CH_3_CN/H_2_O | 70 %, 50 % | [46] |
| 2-(1-(3,5-bis(trifluoromethyl)phenyl)-4,5-diphenyl-*1H*-imidazol-2-yl) phenol **(IMD)** | 452 | 365 | 1x10^-5^ M | THF/H_2_O | 60 % | [47] |
| Hexaphenylsilole **(HPS)** | - | 365 | 1x10^-6^ M | THF/H_2_O | 90 % | [48] |
| 1,1,2,3,4,5-Hexaphenylsilole **(HPS)** and 1-methyl-1-(4-carboxystyrene)-2,3,4,5-Tetraphenylsilole (**MCSTPS)** | - | 365 | 0.1346 g/L and 0.1366  g/L | Ethanol/H_2_O | 60 % and  60 % | [49] |
| 2-[1-(*9H*-fluoren)-4,5-diphenyl-*1H*-imidazol-zyl **(FDIP)** | 450 | 365 | 1x10^-5^ M | Ethanol/H_2_O | 70 % | Present work |

Table S2. Literature of the various AIE materials used for LFPs visualization.

| **Sl.**  **No** | **Material** | **Synthesis procedure** | **Method of LFP detection** | **Types of substrate** | **LFP detection level** | **Aging study** | **References** |
| --- | --- | --- | --- | --- | --- | --- | --- |
|  | 3,4,9,10-perylenetetracarboxylic dianhydride (PTCDA), 1-bromobutane, 1-bromohexane, 1-bromododecane, 1-butanol, 1-hexanol and 1-dodecanol | One-pot etherification reaction | Powder dusting | Non-porous | Type I and II | Up to 3 days | [50] |
|  | TPA-1OH, 4- (diphenylamino)benzaldehyde, | Hydrophilic−hydrophobic molecular architecture | Solution method | Non-porous and porous | Type I, II and III | - | [44] |
|  | 2-hydroxy-5-(me thyl t hio)-  isophthalaldehyde and 2-hydroxy-5-(methoxy) -  isophthalaldehyde | Schiff base condensation reaction | Powder dusting | Non-porous | Type I, II and III | - | [51] |
|  | Tetraphenylethene-based dye (FLA-1) |  | Powder dusting | Non-porous, porous and semi-porous | Type I and II | - | [41] |
|  | 2-hydroxynaphthalene-1-carbaldehyde, (4-fluorophenyl) acetonitrile | Ultra-  sonication | Powder dusting | Non-porous | Type I, II and III | - | [52] |
|  | salicylaldehyde azine (SAA) and montmorillonite (MMT) | Rotary evaporator | Powder dusting | Non-porous and porous | Type I and II | - | [53] |
|  | pyridoxal hydrochloride, hydrazine hydrate, DMSO | Schiff base condensation reaction | Solution method | Non-porous | Type I and II | - | [54] |
|  | Acridinediones | ---------- | Powder dusting | Non-porous | Type I and II | - | [42] |
|  | 3,5-bis-trifluoro-methyl aniline, benzil, ammonium acetate, salicylaldehyde | Acid catalyzed five-member N-heterocyclic ring forming reaction | Powder dusting | Porous | Type I, II and III | - | [47] |
| 10. | Tetrahydrofuran (THF) | Multistep reaction route | Powder dusting | Non-porous, porous and semi-porous | Type I and II | - | [55] |
| 11. | R6G, phenyltrimethoxysilane and MB dyes | Ultrasonication | Powder dusting | Non-porous | Type I and II | - | [56] |
| 12. | tetraphenylethene (TPE) | Wet chemistry treatment | Powder dusting | Non-porous, porous and semi-porous | Type I and II | - | [57] |
| 13. | 2,7-Bis[9,9′- bis(6″-bromohexyl)fluorenyl]-4,4,5,5-tetramethyl-[1.3.2]- dioxaborolane, 4-bromobenzophenone | Wet chemistry treatment | Powder dusting | Non-porous | Type I, II and III | - | [45] |
| 14. | 1,4- dibromonaphthalene (DN) and 1,3,6,8-tetrabromopyrene (TBP), covalent organic polymers (COPs) | Nicatalyzed Yamamoto-type coupling reaction | Spraying solution | Non-porous, porous and semi-porous | Type I, II and III | - | [58] |
| 15. | 2-[1-(*9H*-fluoren)-4,5-diphenyl-*1H*-imidazol-zyl | Acid catalysed | Spraying solution | Non-porous, porous and semi-porous | Type I, II and III | 3 months | Present work |
|  |  |  |  |  |  |  |  |

Table S3. Crystal data and structure refinement of the prepared FDIP molecule.

| **Parameters** | **Data** |
| --- | --- |
| Empirical formula | C34 H24 N2 O |
| Formula weight | 476.55 |
| Temperature | 296(2) K |
| Wavelength | 0.71073 Å |
| Crystal system | Monoclinic |
| space group | P 21/c |
| Unit cell dimensions | a = 14.1603(10) Å  b = 12.6008(8) Å  c = 15.1646(10) Å |
| α | 90 deg. |
| β | 114.591(2) deg. |
| γ | 90 deg. |
| Volume | 2460.4(3) A^3^ |
| Z, Calculated density | 4, 1.286 Mg/m^3^ |
| Absorption coefficient | 0.078 mm^-1^ |
| F(000) | 1000 |
| Crystal size | 0.180 x 0.150 x 0.140 mm |
| Theta range for data collection | 1.582 - 30.690 deg. |
| Limiting indices | -20<=h<=20, -18<=k<=18, -21<=l<=21 |
| Reflections collected / unique | 42107 / 7585 [R(int.) = 0.0410] |
| Completeness to theta = 25.242˚ | 100.0 % |
| Refinement method | Full-matrix least-squares on F^2^ |
| Data / restraints / parameters | 7585 / 0 / 335 |
| Goodness-of-fit on F^2^ | 2.363 |
| Final R indices [I>2sigma(I)] | R_1_ = 0.1431, wR_2_ = 0.4004 |
| R indices (all data) | R_1_ = 0.1919, wR_2_ = 0.4225 |
| Extinction coefficient | n/a |
| Largest diff. peak and hole | 2.516 and -0.697 e. Å^-3^ |
